# Volunteering and Friendship in Later Life: Does Gender Moderate the Relationship?

**DOI:** 10.1093/geroni/igaa057.1341

**Published:** 2020-12-16

**Authors:** Emily Lim, Changmin Peng, Jeffrey Burr

**Affiliations:** University of Massachusetts Boston, Boston, Massachusetts, United States

## Abstract

Friendship, which is an essential part of social life and beneficial to individuals’ well-being, plays a crucial role in maintaining social connectedness in late life. Volunteering is an avenue for older adults to stay socially engaged, and also provides older adults the opportunity to meet and make new friends. A limited literature suggests that volunteering may be associated with friendship, but many studies are limited by reliance on small, non-probability samples and overly simplistic analytic approaches. The literature is also unclear with respect to how volunteering behaviors relate to specific characteristics of friendships and whether there are gender differences underlying these relationships. Using the 2014 wave of the Health and Retirement Study (N=5,306), this study investigates the association between volunteering characteristics (i.e., volunteer status and hours) and friendship characteristics (i.e., the number of close friends, friendship quality, and contact frequency) among community-dwelling older adults. We also examine whether gender moderated these relationships. Results from linear regression analyses indicate that volunteer status and the number of volunteer hours were positively associated with each dimension of friendship. Also, the positive association between volunteering at 1–99 hours, 100–199 hours, and 200 hours or more and number of close friends, friendship quality, and contact frequency were stronger for older women than for older men. Findings demonstrated that volunteering is integral in shaping late-life friendships. The differential benefits of volunteering between older men and women also suggest that volunteering might be more critical for older women’s friendships.

